# Effects of cooking technique and meat cut on the physical and thermal characteristics of camel (*Camelus dromedarius*) meat

**DOI:** 10.5713/ab.25.0566

**Published:** 2025-10-22

**Authors:** Bandar Alfaifi, Abdullah N. Al-Owaimer, Moath B. Othman, Esam H. Fazea, Mansour Ibrahim, Mohamed Shady, Ronnel B. Fulleros, Gamaleldin M. Suliman

**Affiliations:** 1 Department of Agricultural Engineering, College of Food and Agriculture Sciences, King Saud University, Riyadh, Saudi Arabia; 2 Department of Animal Production, College of Food and Agriculture Sciences, King Saud University, Riyadh, Saudi Arabia

**Keywords:** Camel Meat, Electric Oven, Majaheem, Pressure Cooker, Sous vide

## Abstract

**Objective:**

This study aimed to evaluate the effects of cooking methods (COMs) and meat cuts (MTCs) on the physical and thermal characteristics of camel (*Camelus dromedarius*) meat.

**Methods:**

Thirty-two samples from shoulder and round cuts of eight male Majaheem breed animals were prepared using three COMs: sous vide, electric oven, and pressure cooker.

**Results:**

COM had significantly influence on pH, water activity, cooking loss, density, and all color parameters (L*, a*, b*, chroma, hue). MTC significantly affected pH, water activity, cooking loss, and lightness (L*). Both factors significantly impacted specific heat capacity, thermal conductivity, and thermal diffusivity, while thermal resistance was unaffected by MTC. Significant interaction effects (COM×MTC) occurred for pH, thermal diffusivity, and all color parameters except chroma. Sous vide cooking demonstrated superior tenderness and moisture retention compared to electric oven and pressure cooker methods. It effectively preserved natural color attributes, resulting in enhanced lightness (L*), redness (a*), and chroma due to its uniform heating process. This controlled heat distribution minimized moisture loss and improved the meat’s ability to absorb heat, as indicated by elevated specific heat values. These properties contributed to enhanced texture preservation and overall meat quality.

**Conclusion:**

The findings suggest that adopting sous vide could optimize the cooking of camel meat, enhancing its visual appeal and consumer satisfaction.

## INTRODUCTION

Camel meat (*Camelus dromedarius*) is a vital source of nutrition in many regions, especially where camel populations are plentiful, such as the Middle East, North Africa, and Central Asia [1–3]. It offers significant nutritional benefits, being a rich source of protein with levels comparable to other meats [4,5]. It generally has lower fat and cholesterol content than meats like beef, sheep, goat, and chicken, making it a potentially healthier option [5,6]. Camel meat is also rich in essential minerals like iron, zinc, calcium, and magnesium, and contains B vitamins [5]. Some studies suggest that it possesses remedial effects for conditions such as hyperacidity, hypertension, and respiratory diseases [5,6]. Furthermore, its sustainability in arid environments and versatile culinary applications add to its appeal [6]. Despite its nutritional benefits, high protein content, low fat, and essential vitamins and minerals, camel meat has not achieved the same global consumption levels as other meats like beef and lamb. As of 2023, the Saudi Ministry of Environment, Water, and Agriculture estimated the camel population in Saudi Arabia at around 2.2 million, accounting for approximately 5.2% of the world’s camel population [7].

For decades, cooking techniques have been essential for preparing food safely, palatably, and nutritiously [8–10]. Traditional methods include roasting, frying, boiling, steaming, smoking, braising, stewing, and open-fire cooking [10–12]. Advanced techniques encompass precision cooking like sous vide (SOV), flambé, and molecular gastronomy [11,13–15]. Recently, there has been growing interest in strategies promoting energy efficiency and environmental sustainability while maintaining high-quality products [9,16]. These approaches aim to preserve food’s nutritional and sensory properties while minimizing ecological impact [11,17,18]. Techniques such as electric ovens (EOVs), pressure cookers, and SOV enhance meat quality while conserving energy and ensuring safety [11,16,18,19]. EOVs utilize convection, conduction, and radiation for efficient cooking. Convection heats air with elements, circulating it around the meat for even cooking. Conduction occurs when meat contacts a hot surface, transferring heat directly. Radiation from heating elements emits infrared waves that cook the meat’s outer surface [2,20,21]. These mechanisms work together to enhance the meat’s texture and flavor. In contrast, pressure cookers use pressure and steam to elevate cooking temperatures. They create a sealed environment that raises the boiling point of water, allowing steam to penetrate the food, accelerating cooking and tenderizing the meat [18,22]. The SOV method, considered more advanced, involves heating water to a specific temperature in a bath, with meat vacuum-sealed in bags submerged in the water. Heat transfers through conduction, allowing even and gentle cooking over time [2,23]. This slow process preserves the food’s natural flavors, textures, and nutrients, resulting in consistently tender dishes.

In Saudi Arabia, pressure cooking (PRC) is the traditional method for preparing camel meat in all related dishes, while the use of EOVs is limited. Conversely, SOV is not typically considered for camel meat. However, this suggests that SOV could be a valuable alternative cooking method (COM), as it has the potential to produce high-quality dishes while preserving the meat’s nutritional value and desirable eating qualities. The COM used significantly impacts the final product, and the type of meat cut (MTC) plays a crucial role in determining the most suitable method. This relationship affects key quality parameters and organoleptic characteristics, such as tenderness, flavor, juiciness, and texture. Different COMs interact with MTCs in various ways, influencing the overall outcome [24,25]. This study examines how various COMs, specifically SOV, EOV, and PRC, affect the physical and thermal properties of camel meat, focusing on two cuts: shoulder and round. The aim is to understand how these cooking techniques influence the characteristics of cooked camel meat and to identify the most efficient and effective preparation methods.

## MATERIALS AND METHODS

### Preparation of meat cuts and samples

Camel meat samples from eight male animals of the Saudi Arabian Majaheem breed were sourced within 24 hours of slaughter in February 2025. Sixteen samples were taken from the shoulder cut (SC) and round cut (RC). To ensure consistency, the experimental animals were 11–12 months old and had similar nutritional backgrounds. They were provided with similar commercial and traditional basal diets, including barley grains, alfalfa hay, and wheat straw as the main ingredients. Additionally, they were raised on the same farm and subjected to uniform dietary management practices. The samples were transported in an isothermal box with ice packs. Upon arrival at the Food Process Engineering Laboratory at King Saud University, outer fat and connective tissue were removed. The meat was sliced parallel to the muscle fibers into uniform pieces measuring 4 cm×4 cm×2 cm, yielding 32 samples for each cut. These samples were sealed in plastic bags and stored at −20°C until experiments began. Prior to cooking, they were thawed for 24 hours at 3°C in a refrigerator [23,26]. After thawing, the samples were cooked using various methods. This study adhered to ethical guidelines set by the Research Ethics Committee (REC) of King Saud University, classified as noninvasive, utilizing commercially obtained animal tissues without involvement in raising or slaughtering processes.

### Cooking methods

#### Sous vide cooking

Camel meat samples were prepared by sealing the meat portions in vacuum-sealed plastic pouches, specifically designed for SOV cooking. These pouches (Sous-Vide Supreme; Eades Technology of Appliance) have a thickness of 85 μm and are semi-transparent, constructed from multiple layers of food-grade polyethylene and nylon. The materials used for these bags have undergone third-party testing and have been certified to be free of bisphenol A (BPA) and phthalates, ensuring their safety for food contact. The vacuum sealing process was conducted using a Sous-Vide Vacuum Sealer (VS3000; Eades Technology of Appliance), ensuring an airtight seal to maintain optimal cooking conditions. The meat samples were then subjected to cooking in a Sous-Vide water bath (GFL Water Bath, Model 1083; GFL Technology Laboratory Equipment), ensuring an internal temperature of 72°C [2,20]. The water bath was maintained at a constant temperature of 80°C [27], with the precise thermal conditions continuously monitored using a Teflon-coated T-type thermocouple probe (Omega Instrument).

#### Electric oven cooking

Camel meat samples were prepared by wrapping each portion individually in aluminum foil to ensure uniform cooking and temperature distribution. The wrapped samples were then subjected to cooking in a commercial electric stainless-steel grilling oven (Electric Oven, 70L, Stainless Steel; ClassPro). The EOV was preheated to 200°C [24], and the meat was cooked until the internal temperature reached 72°C [28]. To accurately monitor the cooking process, the internal temperature of each sample was continuously measured using a thermocouple probe (Ecoscan Temp JKT; Eutech Instruments). The probe was strategically inserted at the center of each sample to ensure precise and consistent thermal monitoring throughout the cooking process.

#### Pressure cooking

Camel meat samples were subjected to cooking in a commercial gas stovetop pressure cooker (TrustPro Stainless Steel Manual Pressure Cooker, 26 cm/8 L; TrustPro), featuring a sealed pressure vessel and an internal sample basket. Before cooking, two liters of water were added to the pressure cooker. The meat samples were cut to the specified size and placed in the internal basket. They were then cooked at 110°C, with the internal temperature of each sample continuously monitored using a T-type thermometer probe (Copper, fixed, Omega Instrument). This probe was carefully inserted into the geometric center of each sample. The meat was cooked until it reached an internal temperature of 72°C [29].

### Physical properties measurements

#### pH

The pH of the camel meat samples was measured after cooking using a Professional Foodcare Portable Meat pH Meter (HI98163; Hanna Instruments). The sensor of the pH meter was carefully inserted into the meat sample to ensure accurate readings. The device displayed the pH value, which was then recorded for analysis [24].

#### Density

The density of the cooked camel meat was measured using the liquid displacement technique with toluene, employing a density balance (PG 203-S; Mettler-Toledo). The results were displayed directly by the instrument [23].

#### Water activity

The water activity (a_w_) of the cooked camel meat samples was measured using an Aqua Lab Device (Series 3; Decagon Devices). A 20 g sample of the cooked camel meat, chosen to represent the overall batch, was homogenized and placed in the sample chamber of the water activity meter [2]. The measurement was conducted at a room temperature of 22°C±2.

#### Cooking loss

The cooking loss of camel meat was calculated by subtracting the cooked weight from the initial weight [30]. A precision balance, accurate to 0.01 g (model 2100/C/2; Radwag Wagi Elektroniczne), was used to measure the weights of the samples. The cooking loss was determined using the formula:


(1) $$\text{Cooking loss }(\%)=\frac{\text{Fresh camel meat (g)}-\text{Cooked camel meat (g)}}{\text{Fresh camel meat (g)}}\times 100$$

#### Color parameters

The color of the cooked camel meat was assessed using a colorimeter (CR400 Chroma Meter, Konica Minolta; Measuring aperture: 8 mm; Illuminant: CIE D65; Observer angle: CIE 2° Standard Observer) at room temperature (22±2°C). The primary color parameters measured included lightness (L*), redness (a*), and yellowness (b*). Additionally, derived color properties such as hue angle (H°) and chroma (C) were determined [2,24]. The measurements were conducted 30 minutes after cooking, with the samples placed in the optical port of the device, and the results were recorded.

#### Thermal properties measurements

The thermal properties of cooked camel meat, including thermal conductivity (k), thermal diffusivity (α), specific heat (Cp), and thermal resistance (R) were measured at room temperature (22±2°C) using a KD2 Pro thermal properties meter (Decagon Devices). The measurements were conducted by inserting the probe parallel to the protein fibers of the camel meat [23].

#### Statistical analysis

The experimental design of this study employed a completely randomized design with a 2-way factorial arrangement (3×2) investigating three COMs (SOV, EOV, and PRC) across two MTCs: shoulder and round. The normality of the data was assessed using the Shapiro-Wilk test, which ensured the assumptions for ANOVA were met. Data analysis was performed using ANOVA within the General Linear Model (GLM) framework, using SPSS ver. 25 (IBM) employed for statistical computations. Results are presented as mean±standard error of mean, and comparisons were made for independent factors as well as interactions. In cases where significant differences (p≤0.05) were found, post hoc analysis using the least significant difference (LSD) test was performed. Correlations and effect sizes between the independent and dependent factors were also analyzed. Effect sizes were classified as follows: trivial (less than 10%), small (10% to 30%), medium (30% to 50%), and large (greater than 50%) [31,32]. On the other hand, correlations are classified as follows: 0% to 19% indicates a very weak correlation, 20% to 39% represents a weak correlation, 40% to 59% signifies a moderate correlation, 60% to 79% denotes a strong correlation, and 80% to 100% indicates a very strong correlation [31,32]. The following statistical model was used:


(2) $${\text{Y}}_{\text{ij}}=\mu +{\text{A}}_{\text{i}}+{\text{B}}_{\text{j}}+{\text{AB}}_{\text{ij}}+{ɛ}_{\text{ijk}}$$

Where: Y_ij_ = dependent variable, μ = overall mean, A_i_ = effects of COM (SOV, electrical oven, and PRC), B_j_ = effects of MTC (shoulder, and round), ɛ_ijk_ = the random error term associated with the observation.

## RESULTS

### Physical characteristics of camel meat

**Figure 1:**
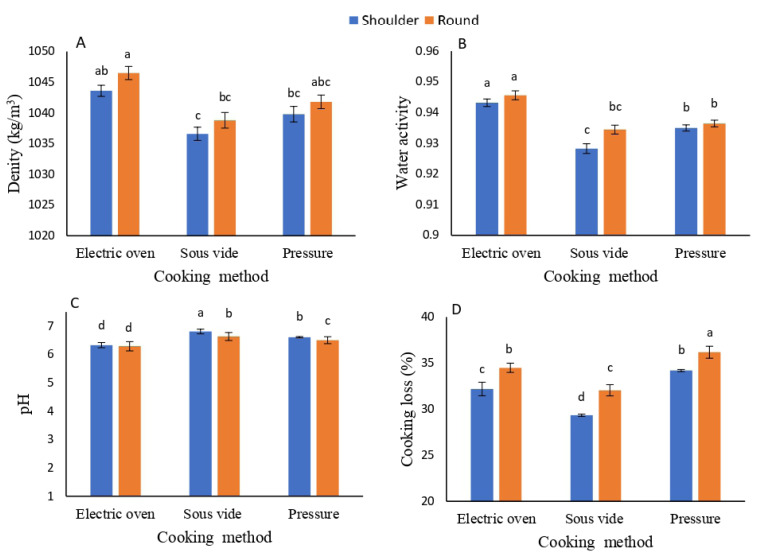
Influence of cooking methods and cut of muscle on the physical properties of cooked camel meat: density (A), water activity (B), pH (C), and cooking loss (%) (D). Results are expressed as the mean±SD (n = 5). ^a–d^ Columns bearing different letters are significantly different at p≤0.05.

Table 1 illustrates the impact of COM and MTC on the physical characteristics of camel meat. Both COM and MTC significantly influenced all studied parameters (p≤0.05), except density. The SOV method yielded the highest pH value (6.72), followed by the stovetop PRC and EOV methods. The SC had a higher pH (6.58) than the RC, which had a pH of 6.47. Density and water activity trends were consistent across the COMs, with EOV showing the highest values (p≤0.001), followed by PRC and then SOV. No statistical difference was observed between RC and SC in terms of density, although RC exhibited the highest value. In contrast, the two MTCs were significantly different in water activity, with RC achieving the highest (p≤0.001) value. Cooking loss was significantly greater (p≤0.001) in camel meat prepared using the PRC method, whereas the SOV method resulted in the lowest water loss during cooking. Additionally, RC exhibited the highest cooking loss (p≤0.001) compared to SC. Notably, the interaction between COM and MTC revealed significant differences (p = 0.03) concerning pH only. Figure 1 presents trends and patterns, simplifying the interpretation of relationships among the variables and enhancing understanding of the data regarding pH, density, water activity, and cooking loss.

### Color components of camel meat and their derivatives

**Figure 2:**
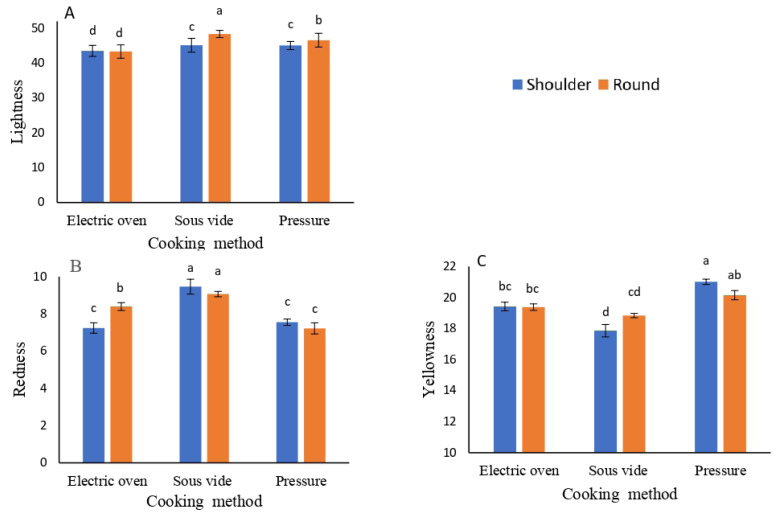
Influence of the cooking method and cut of muscle on the physical properties of cooked camel meat: lightness (A), redness (B), and yellowness (C). Results are expressed as the mean±SD (n = 5). ^a–d^ Columns bearing different letters are significantly different at p≤0.05.

The impact of COM and meat cut (MTC) on the color components of camel meat; lightness (L*), redness (a*), yellowness (b*), and their derivatives chroma (C) and hue (H°) is shown in Table 2. Significant differences (p≤0.001) were found in L*, a*, and b* across COMs. The SOV method significantly affected L* and a*, followed by the stovetop PRC. The b* component was highest with PRC, then EOV, while SOV had the lowest value. SC and RC showed statistically significant difference (p≤0.001) in L* color component only. RC had the highest lightness (L*), while non-significant values for redness (a*) and yellowness (b*). Trends were consistent for color derivatives, with chroma (C) and hue angle (H°) highest in the order: PRC>EOV>SOV. No significant differences were noted between SC and RC for color derivatives; however, RC had higher values. Interactions between COM and MTC were significant (p≤0.001) for all color components except chroma (C). Figure 2 illustrates these effects.

### Correlation and effect size of cooking method and meat cut on the physical characteristics and color components of camel meat

The correlation and effect size of COM and meat cut (MTC) on the physical characteristics of camel meat are shown in Table 3. The pH values related to COM revealed a 38% correlation and a 43% effect size, indicating a moderate positive relationship, meaning 43% of pH variability is explained by COM. Conversely, MTC had an inverse correlation of −20% with pH and a low positive effect size of 7%. Density showed a very low significant (p≤0.05) negative correlation with COM and a minimal positive effect size. Water activity also displayed a very low significant (p≤0.05) negative correlation with COM. In contrast, cooking loss exhibited a very low positive correlation (p≤0.01) and a low effect size (p≤0.05) in response to COM.

The correlation and effect size of COM and meat cut (MTC) on color components of camel meat are presented in Table 3. All color components showed low positive significant correlations with COM, with the highest values in lightness (L*) and redness (a*). Chroma (C) and hue angle (H°) also exhibited very low significant (p≤0.01) positive correlations with COM. Both showed significant positive effect sizes (p≤0.05), though chroma’s value was very low compared to the moderate hue angle. Density, water activity, and cooking loss had very low correlation and effect size values concerning MTC, with only L*, a*, and cooking loss reaching significant levels.

### Thermal characteristics of camel meat

**Figure 3:**
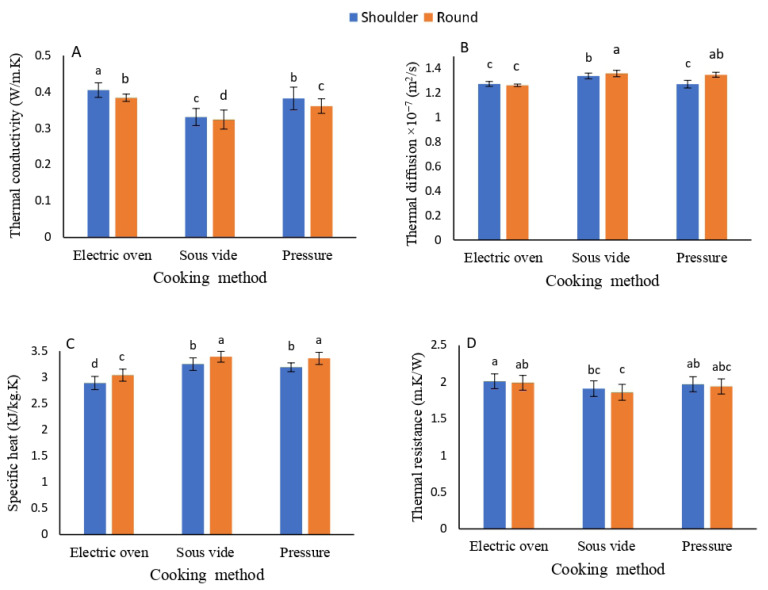
Influence of the cooking method and cut of muscle on the thermal properties of cooked camel meat: thermal conductivity (W/m.K) (A), thermal diffusion (m^2^/s) (B), specific heat (kJ/kg.K) (C), and thermal resistance (m.K/W) (D). Results are expressed as the mean±SD (n = 5). ^a–d^ Columns bearing different letters are significantly different at p≤0.05.

Table 4 presents the effects of COM and meat cut (MTC) on the thermal characteristics of camel meat. All parameters; specific heat, thermal conductivity, thermal diffusion, and thermal resistance showed statistical significance in response to COM and MTC, except for thermal resistance concerning MTC. The SOV method achieved the highest (p≤0.001) values for specific heat and thermal diffusion. In contrast, the EOV recorded the highest (p≤0.001) values for thermal conductivity and thermal resistance. The PRC displayed intermediate values. The RC had the highest (p≤0.001) values for specific heat and thermal diffusion, while the SC excelled in thermal conductivity (p≤0.001). Interactions between COM and MTC revealed significant differences (p≤0.001) in thermal diffusion. Figure 3 illustrates these influences.

### Correlation and effect size of cooking method and meat cut on the thermal characteristics of camel meat

The correlation and effect size of COM and MTC on the thermal characteristics of camel meat are displayed in Table 5. Specific heat and thermal diffusion exhibited a moderate positive correlation (p≤0.001) in response to COM, while thermal conductivity displayed a low positive correlation (p≤0.001). In contrast, thermal resistance showed a non-significant negative correlation with COM. With respect to COM, all thermal characteristics of camel meat exhibited a positive effect size, ranging from low to moderate (p≤0.05). The specific heat and thermal diffusion of camel meat exhibited a positive correlation (p≤0.001) with MTC, though at low levels. In contrast, thermal conductivity and thermal resistance showed low negative correlations, with significance levels of (p≤0.001) and (p≥0.05), respectively. All thermal parameters of camel meat demonstrated a very low significant (p≤0.05) effect size when MTC was considered, except for thermal resistance, which was non-significant.

## DISCUSSION

### Effect of cooking method, and meat cut on the physical characteristics of camel meat

The COM is the primary factor influencing the physical characteristics of camel meat, significantly affecting pH, density, water activity, and cooking loss. Typically, post-mortem muscle breakdown in fresh meat results in a decrease in pH, primarily due to the accumulation of lactic acid. The COMs, however, can modulate this acidification process. Different heat treatments; SOV, EOV, and PRC affect acid production in cooked meat in distinct ways. The experimental results indicate that SOV cooking preserves the pH of meat better than EOV or PRC, likely due to its lower cooking temperatures and controlled heat application, which minimize protein breakdown and acid formation, that helps retain the meat’s natural acidity. Moreover, SOV results in a more tender and less acidic product, likely due to the controlled cooking process that enhances tenderness [33,34]. Additionally, SOV allows for gradual denaturation of muscle proteins, preserving their structure and preventing excessive acid formation [19]. In contrast, EOV involves higher temperatures (200°C), tends to accelerate protein denaturation, leading to more rapid breakdown of muscle fibers. This results in increased acid production and a lower pH in the final cooked product that may affect flavor and texture [11,18]. On the other hand, PRC, uses high temperatures of 110°C combined with steam under pressure. While the steam helps retain moisture, the high temperature promotes the breakdown of muscle proteins, resulting in increased acid production. Pressured-cooked meat showed an intermediate pH value (6.56) between the SOV and EOV methods. The interaction of pressure and heat likely facilitates the production of more acids compared to SOV but not to the extent of EOV cooking. Each method thus influences the acidity and flavor profile of the meat in unique ways.

Density differences indicate that SOV cooking results in a less dense product compared to EOV and PRC. The lower temperatures used in SOV may lead to reduced moisture loss, resulting in a more tender texture [2,23]. In contrast, EOV and PRC typically produce denser products due to greater moisture evaporation. Additionally, variations in water activity demonstrate how cooking temperatures influence moisture retention. SOV cooking retains more water, as indicated by lower water activity values, which are essential for preserving meat quality. Higher water activity can increase the risk of microbial growth, making moisture retention critical [35,36]. Cooking loss values varied significantly, with PRC exhibiting the highest loss due to higher temperatures and pressure, resulting in greater moisture evaporation and tougher meat [37,38]. Overall, COM significantly influences camel meat’s physical characteristics, including pH, density, water activity, and cooking loss. SOV produces a more tender and less acidic product due to its controlled cooking process [33,34,39]. Differences in pH, density, water activity, and cooking loss between SC and RC can be attributed to variations in muscle composition, fat content, and connective tissue. The SC, being less actively used, typically contains more fat and connective tissue, contributing to higher moisture retention. It often contains more intramuscular fat (marbling) and different types of muscle fibers. The SC tends to have a higher proportion of Type I fibers (slow-twitch) due to their functional role. They are more oxidative and are designed for endurance. They are typically found in muscles that are used for sustained activities, such as those in the SC, which is involved in more frequent movement. This results in a higher pH, lower density, and lower cooking loss compared to the RC [40]. The greater fat content in the SC helps retain water during cooking, leading to lower water activity, while the leaner RC has more rapid moisture loss, resulting in higher cooking loss and water activity [41,42]. Studies confirm that pH is essential for meat quality, influencing tenderness and flavor [2].

### Effect of cooking method, and meat cut on the color components of camel meat

The relationship between COM, meat color, and consumer acceptance is shaped by how different techniques affect texture, flavor, and moisture. While fresh meat is typically red, cooking changes its color to brown, signaling doneness and safety. Consumer preferences vary, with bright red favored for raw dishes and brown expected for cooked meat, influencing perceptions of quality and acceptance. On the other hand, the effects of MTC on color are influenced by several factors, including muscle fiber composition, fat content, age and breed of the animal, preparation method, and storage conditions. Cuts from more active muscles tend to be darker due to higher myoglobin levels, while those with more marbling may appear lighter and more appealing. Additionally, older animals generally produce darker meat, and different breeds can exhibit distinct color characteristics. The COM can also affect the final color, as lean cuts may brown more quickly. Finally, storage and handling practices impact color by altering oxygen exposure, which can change the meat’s hue. SOV resulted in the highest lightness (L*), followed by PRC, while EOV had the lowest lightness, attributed to lower cooking temperatures [43]. SOV involves cooking meat in vacuum-sealed pouches at temperatures below 100°C, minimizing the Maillard reaction that causes browning, thus resulting in lower lightness values [2,44]. In contrast, EOV leads to lower lightness due to rapid heating and more pronounced Maillard reactions, which form brown pigments and darken the meat [45]. Studies indicate that faster COMs significantly reduce lightness, as observed with EOV in this study [22]. The redness (a*) of meat is influenced by myoglobin content, providing its red appearance. SOV exhibited the highest redness value, suggesting better retention of red color in camel meat [2,43]. This is likely due to the lower cooking temperatures used in SOV, preventing myoglobin breakdown [33]. EOV resulted in the lowest redness due to higher temperatures that denature myoglobin, making the meat paler. PRC, which combines high heat and moisture, produced moderate redness. The moisture helps preserve myoglobin, but high heat can diminish redness [45].

Yellowness (b*) is a key quality indicator, reflecting fat content, freshness, and the animal’s diet, influencing consumer preferences. Additionally, the b* value indicates the yellow-blue color component, with higher values representing a more intense yellow appearance. Generally, COMs enhance yellowness, with higher fat content contributing to a brighter appearance. In this study, PRC showed the highest yellowness, followed by EOV, while SOV had the least. Increased yellowness in PRC results from fat breakdown and high temperatures, which melt fat and enhance the yellow appearance [22]. SOV retained more moisture, leading to lower yellowness compared to higher-heat methods, with EOV yielding intermediate yellowness values. Interestingly, although the EOV operates at a higher temperature (200°C) compared to the PRC, its b* value is not the highest. This may be attributed to greater moisture loss, leading to drier food that affects color, as well as the dominance of the Maillard reaction, which can produce darker, less vibrant colors. Additionally, in PRC, fats remain evenly distributed and melted, contributing positively to the yellow hue, while in an EOV, fats may separate or oxidize differently, impacting the overall color.

Chroma measures color saturation, with the PRC achieving the highest chroma, followed by the EOV and SOV methods. The pressure cooker’s ability to retain moisture and heat contributed to its vibrant color. In contrast, SOV’s gentler COM resulted in lower chroma due to minimal pigment changes. The EOV produced moderate chroma, effectively balancing moisture loss with pigment retention.

The hue angle measures color tone, with higher values indicating a redder color. PRC had the highest hue angle, followed by EOV and SOV, suggesting PRC yields a color closer to red, while SOV results in a more neutral hue. These findings indicate that COMs retaining moisture and using lower heat, like PRC and SOV, preserve a redder hue in meat [2]. The RC exhibited significantly higher lightness compared to the SC, likely due to differences in fat content and muscle fiber composition [24]. However, no significant differences in redness, yellowness, chroma, or hue angle were observed between the two cuts, indicating that MTC had less impact on these color components compared to the COM.

The interactions between COM and MTC for lightness, redness, and yellowness indicate that COM’s effect on color is influenced by MTC. RC may show different color responses than SC due to variations in muscle structure and fat content, highlighting the complexity of color development in meat and its processing. The results show a significant interaction between the COM and the MTC on the color components and their derivatives. This interaction highlights the need to consider both the COM and the type of MTC when assessing color changes in camel meat. Specifically, the relationship between these two variables indicates that color changes are affected by muscle structure, fat content, and external factors such as cooking temperature, method, and duration [2,11].

### Correlation and effect size of cooking method and meat cut on physical characteristics and color components of camel meat

Correlation measures the strength and direction of the relationship between two or more variables, indicating how closely they change together. A positive correlation means the variables tend to rise or fall together, while a negative correlation suggests they move in opposite directions. Effect size, on the other hand, quantifies the magnitude of the difference between groups or the strength of the relationship between variables. The 38% correlation between pH and COM indicates a weak positive relationship, implying that as COM changes, pH tends to follow. This suggests a small but reliable connection. Alfaifi et al [11] noted that COMs affect pH values in meat, potentially linked to heating rates. The effect size shows that 43% of pH variability can be attributed to COM, indicating a medium effect size and a meaningful influence on camel meat’s pH. Oz et al [25] also found significant effects of COMs on beef pH, supporting these findings. Other physical parameters of camel meat showed weak correlations with COM, while density and water activity exhibited negative correlations, suggesting a reliable but minimal effect. Further research may be needed to understand these dynamics fully, as additional factors could significantly impact the physical properties of camel meat. Several studies [2,24,46] have explored the effects of COMs on cooking loss in various meats, finding significant differences based on the cooking procedures. The correlation between meat texture characteristics (MTC) and camel meat’s physical properties ranged from non-significant to weakly significant. Notably, pH displayed a statistically significant negative weak correlation, indicating that COM significantly influences these characteristics, albeit with minor effects. All physical traits of camel meat showed a positive but trivial effect size with MTC. Only pH, lightness (L*), redness (a*), and cooking loss exhibited statistically significant differences, while yellowness (b*), density, and water activity showed non-significant differences. This suggests a slight tendency for these traits to increase with different MTCs, but the relationship remains minimal. Gök et al [47] reported a significant impact of COM on color components in cattle, primarily due to the Maillard reaction and factors like caramelization, protein denaturation, and moisture loss. Higher cooking temperatures enhance these effects, while weak effect sizes indicate that changes in MTC have little practical significance for these properties. Overall, MTC does not appear to meaningfully impact yellowness, density, or water activity.

### Effect of cooking method, and meat cut on the thermal properties of camel meat

Both COM and meat cut (MTC) significantly impact the thermal properties of camel meat, affecting heat transfer efficiency and final meat quality. Key thermal properties of meat include specific heat, thermal conductivity, thermal diffusion, and thermal resistance, all of which vary depending on the COM and MTC used [11]. COM and MTC influence thermal conductivity, with EOV exhibiting the highest conductivity due to cooking temperatures around 200°C. This rapid heat transfer is facilitated by a greater temperature gradient between the meat’s inner and outer portions [18]. In contrast, SOV operates at lower temperatures (around 80°C), resulting in lower thermal conductivity due to its slow, controlled approach [23]. On the other hand, PRC, at 110°C, accelerates heat transfer using pressure but is less efficient than EOV for rapid heat penetration. The difference in thermal conductivity between SCs and RCs is attributed to their varying muscle compositions [11,18]. Thermal diffusion describes how heat distributes within the meat. SOV achieves superior thermal diffusion, ensuring a more uniform temperature gradient and consistent cooking results, which enhances tenderness. Pressure cooker also achieves high diffusion rates due to the pressurized steam environment, allowing for faster, though less uniform, heat distribution. EOV’s rapid heating of outer layers results in a lower diffusion rate, as the interior heats more slowly [11,18]. The lack of significant differences in thermal diffusion between SC and RC can be also attributed to their similar muscle structures, fat distribution, and moisture content. Both cuts may exhibit comparable thermal properties due to these factors, especially when prepared at similar thicknesses. As a result, these similarities lead to negligible differences in thermal diffusion rates. The higher specific heat in SOV allows meat to absorb more heat without a rapid temperature increase. In contrast, EOV’s higher temperatures lead to quicker cooking and less energy for temperature increases. Pressure-cooked meat shows specific heat values that fall between those of SOV and EOV. This suggests that the pressurized environment of PRC enhances the meat’s ability to absorb and retain heat effectively [18].

The RC, being leaner than the SC, has a higher specific heat due to the properties of its muscle fibers. In contrast, the SC exhibits a lower specific heat because its higher fat content reduces its capacity for thermal energy storage [23,40]. Thermal resistance measures a material’s ability to resist heat flow and is highest in SOV, indicating slower heat transfer at lower temperatures. Conversely, EOV shows lower thermal resistance, allowing for immediate heat penetration. PRC’s thermal resistance indicates that, while heat transfers quickly due to steam, higher moisture content presents some resistance. No significant differences in thermal resistance were observed between SC and RC, suggesting that thermal resistance is more influenced by COM than by specific MTC. Overall, thermal properties such as specific heat, thermal conductivity, thermal diffusion, and thermal resistance vary significantly depending on the COM and MTC. SOV consistently demonstrates higher thermal diffusion and specific heat, indicating optimal heat distribution and retention. In contrast, the EOV exhibits the highest thermal conductivity but less uniform heat diffusion compared to SOV. The PRC shows intermediate results, with high conductivity and diffusion rates due to steam pressure. Additionally, the SC generally has higher thermal conductivity, while the RC exhibits higher specific heat, reflecting differences in fat content and muscle composition.

### Correlation and effect size of cooking method and meat cut on the thermal characteristics of camel meat

The specific heat, thermal conductivity, and thermal diffusion of camel meat showed a statistically significant positive correlation with COMs. The correlation with specific heat was medium, while it was weak for thermal diffusion and very weak for thermal conductivity, indicating that COM notably influences these properties. Conversely, thermal resistance displayed a non-significant, very weak negative correlation with COM, suggesting that it is not directly impacted by COMs. The effect size values indicate varying degrees of influence: medium for specific heat and thermal diffusion, and small for thermal conductivity. Regarding meat type changes, specific heat and thermal diffusion demonstrated weak but statistically significant positive correlations with MTC, indicating slight increases in these properties. However, thermal conductivity showed a very weak negative correlation with MTC, and thermal resistance displayed negligible practical relevance. Overall, while MTC influences some thermal characteristics, the relationships are weak and of limited practical importance.

## CONCLUSION

This study investigates the effects of various COMs: SOV, EOV, and PRC, as well as MTCs, on the physical and thermal properties of camel meat. Among these methods, SOV consistently produces the best outcomes in terms of tenderness and moisture retention, while simultaneously preserving the meat’s natural color and enhancing its lightness, redness, and chroma. In contrast, PRC tends to increase yellowness and hue angle, giving the meat a more intense reddish color. The SOV method can be effectively used in various settings. In restaurants, it ensures consistent quality and allows for efficient meal preparation, enhancing menu innovation. For home cooks, it simplifies cooking and promotes healthier meal options through precise temperature control. Additionally, in large-scale meat production, it standardizes quality, extends shelf life, and facilitates the development of new gourmet products. These applications highlight the method’s adaptability and significant value to industry practitioners. The significant interaction between COM and MTC further highlights the complexity of color development, emphasizing the need for tailored cooking techniques. These results underscore that both COM selection and anatomical cut are critical determinants of final camel meat quality, directly influencing its visual appeal and consumer acceptability. Limitations include the exclusive focus on camel meat, which restricts the generalizability of the results to other meat types. Additionally, the study was conducted under controlled conditions, which may not entirely reflect real-world cooking scenarios as other potential confounding factors and environmental conditions. While the study demonstrated that COMs significantly influence pH and certain physical characteristics, further research is needed to better understand their broader effects on other physical traits and meat quality. The outcomes of this study emphasize the potential for employing advanced and innovative techniques to enhance camel meat quality. This advancement could broaden its availability to consumers globally, thereby improving market reach and increasing consumer satisfaction. Moreover, the insights gained from the study on camel meat can be applied to other types of meat, such as beef, mutton, goats, and poultry, by using similar SOV techniques to enhance tenderness and flavor. Additionally, these principles can inform various COMs, including smoking, PRC, and slow cooking, to optimize moisture retention and flavor extraction. The study’s findings can also inspire culinary innovations, such as fusion cuisine and health-conscious recipes, showcasing the versatility and broader implications of SOV across the culinary landscape.

## Figures and Tables

**Table 1:** Effect of cooking method (COM) and meat cut (MTC) on the physical characteristics of camel meat (N = 32)

Parameter	pH	Density (kg/m^3^)	Water activity	Cooking loss (%)
Cooking method				
Sous vide	6.72^a^	1,037.23^b^	0.931^c^	30.69^c^
Electric oven	6.31^c^	1,045.23^a^	0.944^a^	33.35^b^
Pressure cooker	6.56^b^	1,040.79^c^	0.936^b^	35.19^a^
SEM	0.017	1.46	0.002	0.401
p-value	<0.001	<0.001	<0.001	<0.001
Meat cut				
Shoulder	6.58^a^	1,040.13	0.935^b^	31.91^b^
Round	6.47^b^	1,042.34	0.939^a^	34.24^a^
SEM	0.014	1.19	0.001	0.327
p-value	<0.001	NS	0.03	<0.001
Interaction				
COM×MTC	0.03	NS	NS	NS

a–c Means on the same column with different superscripts are significantly different at p≤0.05.

SEM, standard error of mean; NS, non-significant.

**Table 2:** Effect of cooking method (COM) and meat cut (MTC) on the color components (lightness L*, redness a*, and yellowness b*) and their derivatives C and H^0^ of camel meat (N = 32)

Parameter	L*	a*	b*	C	H^0^
Cooking Method					
Sous vide	46.74^a^	9.26^a^	18.89^c^	19.60^c^	63.86^c^
Electric oven	43.42^c^	7.82^c^	19.40^b^	20.96^b^	67.45^b^
Pressure cooker	45.81^b^	8.38^b^	20.58^a^	21.92^a^	69.98^a^
SEM	1.46	1.12	0.26	0.12	0.30
p-value	<0.001	<0.001	<0.001	0.001	<0.001
Meat cut					
Shoulder	44.56^b^	8.08	19.40	21.11	62.21
Round	46.09^a^	8.23	19.47	21.20	66.97
SEM	0.27	0.09	0.21	0.21	0.25
p-value	<0.001	NS	NS	NS	NS
Interaction					
COM×MTC	<0.001	0.001	0.04	NS	<0.001

a–c Means on the same column with different superscripts are significantly different at p≤0.05.

SEM, standard error of mean; NS, non-significant.

**Table 3:** Correlation and effect size of cooking method (COM) and meat cut (MTC) on the physical characteristics and color components and their derivatives of camel meat

Parameter	Cooking method		Meat cut	

	Correlation (%)	Effect size (%)	Correlation (%)	Effect size (%)
pH	38^**^	43^*^	−20^**^	7^*^
Density (kg/m^3^)	−15^*^	7^*^	9	1
Water activity	−26^**^	16^*^	12	2
Cooking loss (%)	15^**^	15^*^	24^*^	7^*^
Lightness (L*)	24^**^	13^*^	19^**^	4^*^
Redness (a*)	12^*^	29^*^	5	8^*^
Yellowness (b*)	16^**^	9^*^	1	1
Chroma (C)	13^**^	5^*^	1	1
Hue angle (H^0^)	17^**^	40^*^	−3	1

* and ** indicate that the correlation coefficient and effect-size ratio are significant at p≤0.05 and 0.01, respectively.

**Table 4:** Effect of cooking method (COM) and meat cut (MTC) on the thermal characteristics of camel meat (N = 32).

Parameter	Specific heat (kJ/kg.K)	Thermal conductivity (W/m.K)	Thermal diffusion×10^−7^ (m^2^/s)	Thermal resistance (m.K/W)
Cooking method				
Sous vide	3.32^a^	0.33^c^	1.35^a^	1.89^c^
Electric oven	2.97^c^	0.39^a^	1.27^c^	2.00^a^
Pressure cooker	3.28^b^	0.37^b^	1.32^b^	1.95^b^
SEM	0.01	0.004	<0.001	0.02
p-value	<0.001	<0.001	<0.001	<0.001
Meat Cut				
Shoulder	3.12^b^	0.37^a^	1.30^b^	1.96
Round	3.27^a^	0.35^b^	1.32^a^	1.93
SEM	0.07	0.003	0.03	0.017
p-value	<0.001	<0.001	<0.001	NS
Interaction				
COM×MTC	NS	NS	<0.001	NS

a–c Means on the same column with different superscripts are significantly different at p≤0.05.

SEM, standard error of mean; NS, non-significant.

**Table 5:** Correlation and effect size of cooking method (COM) and meat cut (MTC) on the thermal characteristics of camel meat

Parameter	Cooking method		Meat cut	

	Correlation (%)	Effect size (%)	Correlation (%)	Effect size (%)
Specific heat (kJ/kg.K)	43^**^	31^*^	26^**^	1^*^
Thermal conductivity (W/m.K)	18^**^	28^*^	−16^**^	1^*^
Thermal diffusion (m^2^/s)	35^**^	38^*^	20^**^	1^*^
Thermal resistance (m.K/W)	−8	35^*^	−7	1

* and ** indicate that the correlation coefficient and effect-size ratio are significant at p≤0.05 and 0.01, respectively.

## Data Availability

Upon reasonable request, the datasets of this study can be available from the corresponding author.
